# Estimated five-year survival and direct healthcare costs of adult patients with gastric cancer: real-world evidence from a tertiary hospital in Colombia

**DOI:** 10.3389/fpubh.2026.1820842

**Published:** 2026-06-18

**Authors:** Uriel Palacios-Barahona, Fabio Olivella, Alex Arroyo Santos, Katheryne Valencia Triana, Diego Rojas-Gualdrón

**Affiliations:** 1 Cancer Institute & Hematology, Hospital Universitario Mayor Méderi, Universidad del Rosario, Bogotá, Colombia; 2 School of Medicine, Universidad CES, Medellín, Colombia

**Keywords:** Colombia, costs and cost analysis, hospital costs, stomach cancer, survival analysis

## Abstract

Gastric cancer remains a major cause of cancer mortality worldwide, particularly in middle-income countries, where late diagnosis is frequent and information on its economic impact is limited. We evaluated survival outcomes and direct healthcare costs of adult patients with gastric cancer treated between 2019 and 2024 at Hospital Universitario Mayor Méderi, a tertiary referral hospital in Bogotá, Colombia, using routinely collected clinical and administrative data from a tertiary referral hospital. Overall survival and healthcare resource utilization were described, and cumulative direct medical costs over 5 years were estimated in thousands (k) of 2023 international dollars (Int$). A total of 616 adult patients with gastric cancer were identified in hospital records during the study period. Of these, 388 patients (63.0%) met the eligibility criteria and were included in the final analytic sample, whereas 228 patients (37.0%) were excluded according to the predefined exclusion criteria. The median age was 65 years [interquartile range (IQR): 57–73]; 36.9% women). Using Tumor–Node–Metastasis staging (TNM: tumor extent, regional lymph node involvement, and distant metastasis) recorded in medical charts, 36.3% had stage III and 34.8% stage IV disease. Initial treatment intent was curative in 46.6% and palliative in 53.4%. The estimated cumulative mean direct cost was Int$ 89.3 k (95% CI: 75.9 k–102.8 k) at 2 years and Int$ 161.2 k (95% CI: 92.0 k–230.4 k) at 5 years. Median overall survival was 30.7 months (95% CI: 22.7–38.7), and the estimated 5-year survival probability was 40.0%; it was 45.5% for stage III disease and 12.5% for stage IV disease. Gastric cancer imposes both high mortality and substantial economic burden on the healthcare system, largely determined by the clinical stage at diagnosis. These findings highlight the potential population-level and financial benefits of strategies aimed at earlier detection and timely access to oncologic care in middle-income countries.

## 1 Introduction

Gastric cancer remains a major contributor to the global burden of noncommunicable diseases. According to the Global Burden of Disease (GBD) 2023 study (1), the worldwide age-standardized prevalence and incidence rates were 29.87 and 14.00 per 100,000 population, respectively. It ranked as the 23rd leading cause of disability-adjusted life years (DALYs) among non-communicable diseases and the fourth among neoplasms. Although global mortality has declined by approximately 56% since 1990, reaching 10.28 per 100,000 in 2023, projections suggest that mortality reductions may slow, and years lived with disability may increase by 2050 (2). These epidemiological trends highlight a persistent and evolving burden at the population level.

In Colombia, gastric cancer remains a major public health concern. GLOBOCAN 2022 estimated that gastric cancer was among the leading causes of cancer incidence and mortality in the country, with 8,938 new cases and 6,901 deaths reported in 2022 (3). National administrative data from the High-Cost Diseases Fund also show that cancer represents a growing burden for the health system; in the 2024 reporting period, 15,704 prevalent cancer cases and 2,272 new cases were reported (4). Late diagnosis remains a central challenge, particularly among older adults, in whom multimorbidity and functional status may influence treatment pathways, resource use, and survival outcomes.

Beyond its clinical impact, gastric cancer represents a substantial economic burden. Previous studies have identified it as one of the costliest malignancies because of the productivity losses associated with premature mortality (5, 6). In addition, direct healthcare costs arise from diagnostic procedures, surgical management, systemic therapies, and supportive care (7–9). In the United States, total spending on gastrointestinal cancers has increased substantially, driven by price growth and intensity of care, with projections estimating annual expenditures of USD 21 billion by 2050 (10). However, most cost-of-illness estimates originate from high-income countries, limiting their applicability to middle-income countries.

Colombia has a social health insurance system organized mainly through contributory and subsidized schemes. Despite broad formal coverage for diagnostic, surgical, pharmacological, hospital-based, supportive, and palliative care services, most patients with gastric cancer are diagnosed at stages III–IV, and Tumor-Node-Metastasis (TNM) classification is unknown in 29.17% of cases. The observed 5-year overall survival is 25.15% (95% CI: 24.34–25.96), decreasing from 52.49% in stage I to 6.67% in stage IV, underscoring late diagnosis as a persistent challenge and supporting the need for local evidence on survival and direct healthcare costs (11).

In middle-income settings, such as Colombia, changing epidemiological patterns, technological adoption, and healthcare utilization dynamics may substantially influence both survival outcomes and cost trajectories. In Colombia, gastric cancer is generally managed through stage-based multidisciplinary care, including endoscopic or surgical resection for early disease, multimodal treatment for resectable locally advanced tumors, and systemic therapy with palliative intent for unresectable or metastatic disease. However, real-world evidence on long-term survival and cumulative direct healthcare costs of gastric cancer remains scarce. Generating locally grounded cost-of-illness data is essential for informing resource allocation, budgeting, and strategies aimed at earlier diagnosis and more efficient care delivery. This study aimed to estimate five-year overall survival (OS) and cumulative direct healthcare costs of adult patients with gastric cancer treated between 2019 and 2024 at a tertiary referral hospital in Bogotá, Colombia, using routinely collected clinical and administrative data.

## 2 Materials and methods

### 2.1 Study design and settings

This retrospective longitudinal observational study was based on routinely collected real-world clinical and administrative data. Adult patients diagnosed with gastric cancer were followed from the date of diagnosis until death, the last recorded healthcare visit, or the administrative end of available follow-up. Patients diagnosed between January 2019 and December 2024 were eligible. Data extraction and verification were performed during the first quarter of 2025. The study was conducted at Hospital Universitario Mayor Méderi, a tertiary referral teaching hospital in Bogotá, Colombia, affiliated with Universidad del Rosario through academic, clinical, and research activities, including hospital-based and outpatient oncology services, which provides comprehensive oncologic care within the Colombian health system.

### 2.2 Participants

We consecutively included all patients who were (1) aged 18 years or older, (2) had a diagnosis recorded in the institutional electronic health record and confirmed by pathology report, and (3) had less than 1 year between diagnosis and the first healthcare contact at the institution to improve completeness of early treatment and cost capture from the time of diagnosis. We excluded patients with: (1) concomitant cancer; (2) only one medical visit at the institution during the study period, because a single encounter may reflect an initial consultation or incomplete referral rather than sustained institutional oncologic care; or (3) no available data on clinical stage. Potentially eligible patients were identified through administrative record filtering using International Classification of Diseases, 10th Revision (ICD-10) codes for gastric cancer (C16.0–C16.9), and fulfillment of eligibility criteria was manually verified through detailed medical chart review. Follow-up to determine vital status was performed using hospital medical and administrative records and was complemented with the data from the Administrator of the Resources of the General Social Security System in Health (ADRES) when needed.

### 2.3 Clinical variables

Overall survival (OS), defined as the time from diagnosis to all-cause death, was the clinical outcome. Death status was determined using hospital administrative and medical records complemented by a review of the Colombian national social security data system ADRES, which was used to verify vital status and health insurance affiliation when hospital data were incomplete. The clinical and demographic characteristics of patients at the first healthcare visit, including age, sex, educational level, health insurance scheme, the Eastern Cooperative Oncology Group (ECOG) Performance Status Scale, the Charlson Comorbidity Index, clinical stage, type of cancer (adenocarcinoma, squamous-cell carcinoma, stromal tumor, or neuroendocrine tumor), distant metastasis, and initial treatment, were also collected. All clinical data were gathered from medical records. The ECOG Performance Status Scale ranges from 0 (fully active) to 4 (completely disabled). Clinical stage was assigned according to the TNM staging system, which classifies disease extent based on primary tumor extent (T), regional lymph node involvement (N), and distant metastasis (M), as recorded in the medical chart. Stage I indicates localized disease, stages II–III indicate progressively more extensive locoregional disease, and stage IV indicates metastatic disease. Initial treatment was classified according to intent as curative or palliative. Curative-intent treatment included endoscopic or surgical resection, perioperative systemic therapy, and/or adjuvant treatment when indicated. Palliative-intent treatment included systemic therapy, symptom-directed procedures, supportive care, and palliative care for unresectable or metastatic disease.

### 2.4 Economic variables

Costs were estimated from the healthcare provider perspective following a cost-of-illness framework. The direct costs of medical care, total and by inpatient and outpatient settings, were defined as the primary economic variables; including billings from outpatient settings allowed us to capture high-cost oncologic therapies which are relevant drivers of direct cost of care, particularly at locally advanced disease and advanced stages. As secondary variables, we included variables related to service utilization: duration of care (time between first and last healthcare visit), cumulative stay in general ward, intermediate care (IMCU), and intensive care (ICU) units, number of emergency department visits, number of outpatient clinic visits, and number of medical procedures. Medical procedures were defined as diagnostic and therapeutic interventions recorded in administrative billing data, including, but not limited to, central venous catheter placement, biopsies, endoscopic procedures, and other invasive or interventional services.

Inpatient costs included services billed during hospital admissions, including general ward, intermediate care, intensive care, inpatient procedures, medications, and related hospital services. Outpatient costs included services billed without hospital admission, including oncology consultations, chemotherapy or infusion services, ambulatory diagnostic procedures, endoscopic procedures, and outpatient medical procedures.

All economic variables were obtained from administrative records from the Hospital Universitario Mayor Méderi Department of Business Intelligence, including both inpatient and outpatient billing records generated within the institution and its outpatient oncology services. Costs were estimated using a top-down (macro-costing) approach based on hospital billing records from the healthcare provider perspective. Costs were adjusted for inflation using the health sector-specific consumer price index (2019: 2.82%; 2020: 4.96%; 2021: 3.98%; 2022: 9.53%; 2023: 9.49%; 2024: 5.54%) and converted to 2023 international dollars (Int$) using purchasing power parity (PPP), based on the 2023 health services conversion factor of 824 COP/ Int$ (World Bank). All cost values are reported in thousands (k).

### 2.5 Bias

To control for potential selection and information bias, eligibility criteria were verified, and data were gathered from medical records by a nurse research assistant under the supervision of two researchers (KVT and UPB). To reduce biases in the estimation of the cumulative cost of care due to censoring and deaths, the Bang and Tsiatis (2000) method was employed (12), with estimations restricted to time periods where relative precision was ≤20% and censoring rate <20%. Comorbidity burden was measured using the Charlson Comorbidity Index. Detailed multimorbidity patterns and individual comorbid conditions were not modeled separately as cost predictors and were considered in the interpretation of the study limitations.

### 2.6 Study size

This was a retrospective longitudinal observational study based on routinely collected real-world clinical and administrative data. Therefore, no *a priori* sample size calculation was performed. The study included all consecutive adult patients with gastric cancer who met the eligibility criteria and received care at the institution during the study period.

### 2.7 Statistical methods

Clinical and demographic data are described as median and interquartile range (IQR) for quantitative variables and frequency and percentage for categorical variables. Overall survival was analyzed using interval-censored parametric survival regression models. Exact dates of death were treated as observed uncensored events. When only the death notification date (rather than the exact date of death) was available, cases were treated as interval-censored, with the interval defined as the time from the last recorded survival date to the date of death notification. This approach was used to avoid assigning an exact date of death when only an interval in which death occurred could be established. Patients who did not die during follow-up were right-censored. Gompertz distribution was selected over Exponential, Weibull, Lognormal, Loglogistic, and Generalized Gamma as it provided the best fit according to the Akaike and the Bayesian information criteria. Because not all patients had complete 5-year observed follow-up, estimated 5-year survival probabilities were derived from the fitted Gompertz survival function. Median survival time with 95% confidence interval (95%CI) and model-based estimated 5-year survival probabilities are presented for the full sample and by clinical stage. The same Gompertz model was employed to analyze the associated factors. Crude and adjusted hazard ratios (HR) with 95% confidence intervals (CI) were obtained using simple and multiple regression models. Variables were selected for multivariable modeling based on clinical relevance and univariable association (*p* < 0.25).

The observed direct costs and service utilization variables were described using the median and IQR. To properly adjust for censoring and deaths, the time-restricted mean cost (TRMC) function was estimated using the Bang and Tsiatis (2000) method based on the total cost (12). The mean cumulative cost up to 5 years since diagnosis with 95% CI is presented for the full sample. Subgroup analysis by clinical stage is also presented but restricted to 27 and 48 months for patients with stage I-II and stage IV cancer, respectively, due to high censoring, leading to unstable and imprecise (>20%) estimates. Statistical significance was set at *p* < 0.05. No patients were excluded solely because of missing covariate data. Missing ECOG performance status was addressed in sensitivity analysis as described below.

### 2.8 Sensitivity analysis

We performed sensitivity analyses to assess the potential impact of missing ECOG performance status (16.5% missing) and potential immortal time bias on HR estimates. To impute missing ECOG data, we conducted multiple imputation by chained equations (MICE) using an ordered logistic regression model including all covariates from the final multivariable model. We then refit the same final model used in the primary analysis within the imputed datasets and pooled estimates. To evaluate potential immortal time bias, we performed a 30-day landmark analysis using the imputed datasets and refit the same final model from the primary analysis with follow-up redefined from day 30 onward. Bias attributable to missing ECOG data was assessed by comparing HRs from the multiple imputation analysis with those from the complete-case primary analysis, and potential immortal time bias was assessed by comparing HRs from the landmark analysis with those from the primary analysis.

To assess histologic heterogeneity, we conducted an additional sensitivity analysis comparing adenocarcinoma versus gastric stromal tumors, given their distinct biological behavior and treatment pathways. For overall survival, we estimated the association between histologic subtype and mortality using a Cox proportional hazards model. For costs, we compared adjusted median total direct costs using quantile regression, adjusting for length of follow-up, clinical stage, and Charlson comorbidity index.

The analyses were performed using Stata version 16.1 (College Station, TX).

### 2.9 Ethics

The study was approved by the Institutional Research Ethics Committee of Universidad del Rosario, Life Sciences Board (approval number: DVO005 2,542–CV1831; approval date: February 12, 2024). The committee approved the study after expedited review and waived the requirement for informed consent because the study was retrospective and classified as no-risk research, in accordance with Colombian Resolution 8,430 of 1993, Title II, Chapter 1, Article 16, first paragraph. Hospital Universitario Mayor Méderi authorized access to anonymized institutional clinical and administrative data for research purposes. The hospital is affiliated with Universidad del Rosario through academic, clinical, and research activities.

## 3 Results

### 3.1 Participants

A total of 616 adult patients with gastric cancer were identified in hospital records during the study period. Of these, 388 patients (63.0%) met the eligibility criteria and were included in the final analytic sample, whereas 228 patients (37.0%) were excluded according to the predefined exclusion criteria. The median follow-up of 17.2 months (IQR: 7.8 27.6) were included in the analysis. The median age at study entry was 65 years (IQR: 57–73), and 36.9% of the patients were women. Most patients had primary or secondary education (75%) and belonged to the contributive regime (83.8%) of the social security system. In terms of functional status, a third of the patients had ECOG 0 (35.1%) and a third ECOG 1 (34%). The median CCI was 2 (IQR: 1–3; Table 1).

**Table 1:** Medical and demographic characteristics at study entry (*n* = 388).

	n	%
Age at diagnosis, Median (IQR)	65	(57–73)
Woman	143	36.9
Education		
None	56	14.4
Primary or secondary	291	75
Tertiary	40	10.3
No data	1	0.3
Social security		
Contributive	325	83.8
Subsidized	55	14.2
No data	8	2.1
Eastern Cooperative Oncology Group (ECOG)		
0	136	35.1
1	132	34
2	30	7.7
3	22	5.7
4	4	1
No data	64	16.5
Charlson Comorbidity Index†, Median (IQR)	2	(1–3)
Type of gastric cancer		
Adenocarcinoma	367	94.6
Squamous-cell carcinoma	2	0.5
Stromal tumor	18	4.6
Neuroendocrine tumor	1	0.3
Clinical stage		
I	36	9.3
II	76	19.6
III	141	36.3
IV	135	34.8
Documented distant metastatic sites during follow-up	170	43.8
Bone	7	1.8
Central nervous system	1	0.3
Liver	56	14.4
Lungs	8	2.1
Another site	100	25.8
No data	29	7.5
Initial treatment		
Curative	181	46.6
Paliative	207	53.4

Clinical stage was assigned according to the Tumor–Node–Metastasis (TNM) staging system. TNM, Tumor–Node–Metastasis staging system, based on primary tumor extent (T), regional lymph node involvement (N), and distant metastasis (M). Clinical stage I indicates localized disease; stages II–III indicate progressively more extensive locoregional disease; and stage IV indicates metastatic disease. ECOG: 0 = fully active, 1 = restricted in strenuous activity, 2 = ambulatory and capable of self-care but unable to work, 3 = limited self-care, 4 = completely disabled. IQR: interquartile range. Distant metastatic involvement was abstracted from clinical record documentation and may reflect metastatic sites recorded during institutional care; therefore, it is not directly equivalent to clinical stage at diagnosis. Site-specific categories are not mutually exclusive.

Most tumors were adenocarcinomas (94.6%). Regarding disease extent at study entry, 36.3% of patients were classified as stage III and 34.8% as stage IV. Initial treatment intent was curative in 46.6% of patients and palliative in 53.4%.

A total of 388 patients were included in the final analytic sample. In a post-hoc precision assessment, the final sample size provided an acceptable level of precision for the primary estimates, with confidence interval widths considered adequate for the descriptive objectives of the study.

### 3.2 Observed direct cost of care and service utilization

The median annual direct cost of care was Int$ 49.0 k (IQR: 22.6 k–104.7 k), ranging from Int$ 17.7 k (IQR: 10.2 k–62.2 k) for patients with stage I cancer to Int$ 80.8 k (IQR: 40.6 k–188.5 k) for those with stage IV cancer (Table 2). In the inpatient setting, the median annual direct cost of care was Int$ 22.6 k (IQR: 9.1 k–61.7 k), with a median utilization of 14.5 days (IQR: 5.0–26.0) in the general ward. Among patients with stage IV cancer, the median annual inpatient cost was Int$ 31.5 k (IQR: 11.9 k–128.0 k), and the median general ward utilization was 16.0 days (IQR: 8.0–27.0). In the outpatient setting, the median annual direct cost of care was Int$ 12.6 k (IQR: 5.0 k–28.9 k), with a median of 24.0 clinic visits (IQR: 8.0–40.0). Among patients with stage IV cancer, the median annual outpatient cost was Int$16.8 k (IQR: 5.5 k–48.1 k), and the median number of clinic visits was 15.0 (IQR: 2.0–37.0).

**Table 2:** Direct healthcare costs and service utilization by clinical stage (Median and interquartile range).

	Full sample	Clinical stage (Int$)			
		I	II	III	IV
Direct cost of care, annual					
Total, Int$	49.6 (22.6–104.7)	17.7 (10.2–62.2)	31.4 (15.6–55.7)	47.9 (20.8–94.3)	80.8 (40.6–188.5)
Inpatient, Int$	22.6 (9.1–61.7)	12.1 (2.1–43.0)	14.1 (5.3–33.3)	21.7 (9.5–46.8)	31.5 (11.9–128.0)
Outpatient, Int$	12.6 (5.0–28.9)	3.5 (2.5–7.2)	10.8 (4.9–20.3)	14.5 (6.8–28.7)	16.8 (5.5–48.1)
Direct cost of care, total					
Total, Int$	58.2 (28.9–93.9)	37.6 (6.8–79.7)	56.7 (36.2–76.7)	65.4 (35.1–108.9)	53.5 (26.9–108.1)
Inpatient, Int$	32.1 (15.8–55.1)	28.6 (1.7–58.6)	29.3 (15.6–52.1)	36.3 (20.2–56.6)	27.8 (11.5–47.6)
Outpatient, Int$	15.8 (4.0–38.9)	5.9 (2.5–10.6)	19.1 (5.2–36.6)	21.3 (7.1–46.3)	12.8 (1.9–44.8)
Service utilization					
Duration of care, months	17.2 (7.8–27.6)	20.2 (10.5–27.6)	21.8 (10.9–30.5)	19.0 (10.4–30.0)	10.4 (2.0–21.2)
Inpatient					
General ward, days	14.5 (5.0–26.0)	5.0 (0.5–16.5)	9.5 (3.0–20.5)	17.0 (6.0–26.0)	16.0 (8.0–27.0)
IMCU, days†	0.8 (2.3)	1.6 (4.3)	0.7 (1.8)	0.8 (2.0)	0.7 (2.0)
ICU, days†	0.6 (2.8)	2.1 (5.3)	0.8 (4.1)	0.6 (2.1)	0.2 (0.7)
Outpatient					
Clinic, visits	24.0 (8.0–40.0)	20.5 (11.5–28.0)	27.0 (18.0–42.5)	29.0 (14.0–44.0)	15.0 (2.0–37.0)
Procedures, number†	1.9 (3.9)	0.7 (1.1)	1.5 (2.4)	2.0 (4.4)	2.3 (4.4)
Urgency, visits†	1.2 (1.5)	0.9 (1.3)	1.1 (1.5)	1.1 (1.3)	1.4 (1.6)

Clinical stage was assigned according to the Tumor–Node–Metastasis (TNM) staging system. TNM, Tumor–Node–Metastasis staging system, based on primary tumor extent (T), regional lymph node involvement (N), and distant metastasis (M). Clinical stage I indicates localized disease; stages II–III indicate progressively more extensive locoregional disease; and stage IV indicates metastatic disease. IQR, Interquartile range, Int$: Costs are expressed in thousands of 2023 international dollars. IMCU, Intermediate Care Unit; ICU, Intensive Care Unit. All cost values are reported in thousands.

### 3.3 Cumulative direct cost of care

**Figure 1:**
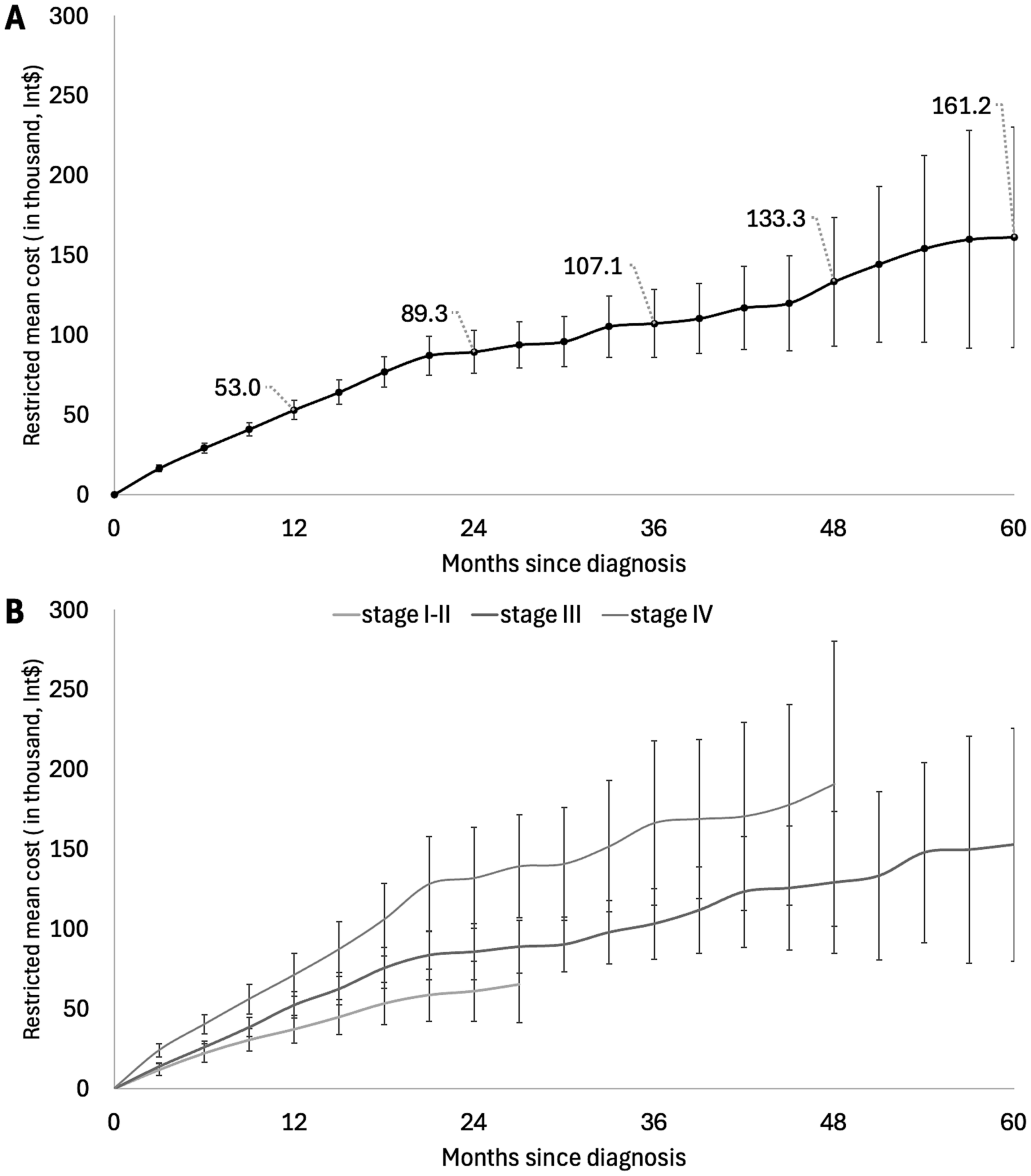
5-year overall survival function (Gompertz distribution) in the full sample **(A)** and by clinical stage at study entry **(B)**.

Figure 1A presents the restricted mean cost function for up to 5 years since diagnosis, with costs expressed in thousands of 2023 international dollars (Int$). For the full sample, cumulative costs increased approximately linearly during the first 24 months, reaching Int$ 89.3 k (95% CI: 75.9 k–102.8 k). Thereafter, the slope flattened, reaching a 48-month cumulative mean cost of Int$ 133.3 k (95% CI: 93.0 k–173.6 k). The estimated five-year total restricted mean cost was Int$ 161.2 k (95% CI: 92.0 k–230.4 k). Figure 1B presents the estimated cumulative mean costs by clinical stage. In an additional analysis by initial treatment intent, the estimated mean cumulative direct cost was Int$ 93.3 k (95% CI: 37.5 k–149.0 k) for patients treated with palliative intent and Int$ 175.1 k (95% CI: 117.3 k–232.8 k) for those treated with curative intent. The estimated mean difference was Int$ 81.8 k (95% CI: 1.6 k–162.1 k; *p* = 0.046), suggesting higher cumulative costs among patients initially managed with curative intent.

### 3.4 Overall survival and factors associated to overall survival

**Figure 2:**
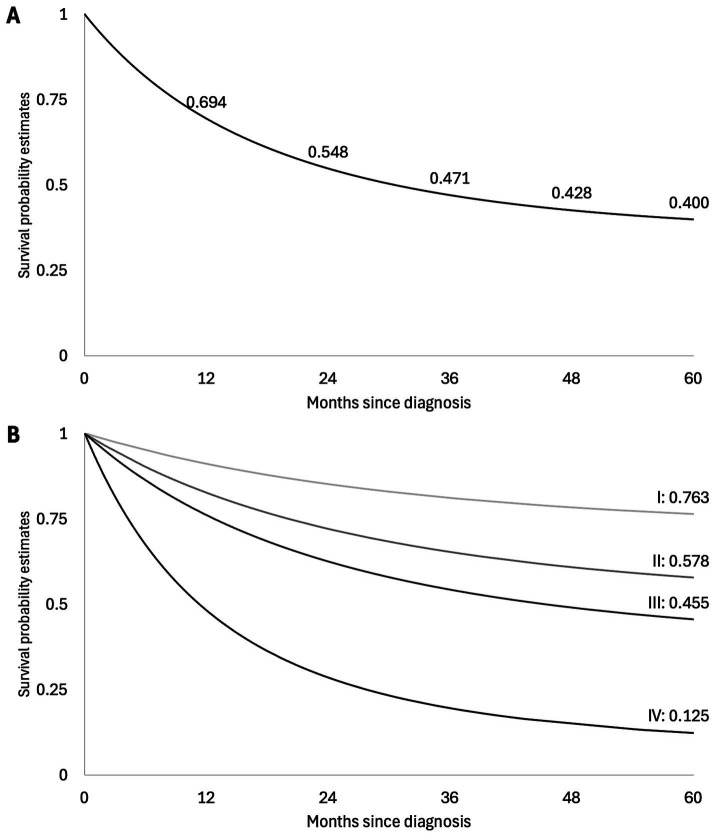
5-year mean cumulative cost function (in thousand Int$) in the full sample **(A)** and by clinical stage **(B)**. Censoring rates in the full sample were 0.29 at 1 year, 0.51 at 2 years, 0.67 at 3 years, 0.77 at 4 years, and 0.78 at 5 years. Estimates for stage I–II and stage III patients are presented up to 27 and 48 months, respectively, due to high censoring and resulting imprecision of the cost function.

The median overall survival was 30.7 months (95% CI: 22.7–38.7), and the Kaplan–Meier estimated 5-year survival probability was 40.0% (Figure 2). The 5-year survival of patients with stage I (76.3%) and stage II (57.8%) was higher than 50%, and the median survival was not estimated. For patients with stage III cancer, the median OS was 41.8 months (95% CI:27.1–56.5) with an estimated 5-year survival probability of 45.5%, and for patients with stage IV cancer, the median OS was 9.9 months (95% CI:7.1–12.8) with an estimated 5-year survival probability of 12.5%.

Table 3 presents the observed and adjusted estimations of the association between clinical characteristics and OS. ECOG performance status and clinical stage showed statistically significant associations. Compared to ECOG 0, patients with functional status ECOG 2 (aHR 2.37; 95% CI: 1.42–3.98), ECOG 3 (aHR 3.88; 95% CI: 2.29–6.58), and ECOG 4 (aHR 7.03; 95% CI: 2.51–19.72) presented lower OS. In relation to clinical stage, compared to stage I, we estimated an aHR of 2.50 (95% CI: 1.06–5.89) for stage III and an aHR of 5.99 (95% CI: 2.57–13.96) for stage IV cancer.

**Table 3:** Patient characteristics associated to overall survival.

	Observed			Adjusted*		
	HR	95%CI	*p*-value	aHR	95%CI	*P*-value
ECOG Performance Status Scale						
0	1.00			1.00		
1	1.42	(0.99–2.04)	0.057	1.44	(0.99–2.09)	0.057
2	2.67	(1.63–4.38)	<0.001	2.37	(1.42–3.98)	0.001
3	4.75	(2.84–7.95)	<0.001	3.88	(2.29–6.58)	<0.001
4	6.41	(2.32–17.68)	<0.001	7.03	(2.51–19.72)	<0.001
Type of gastric cancer						
Adenocarcinoma	4.13	(1.32–12.94)	0.015	3.57	(1.14–11.11)	0.030
Squamous-cell carcinoma	9.20	(0.95–88.76)	0.055	N/E		
Stromal tumor	1.00			1.00		
Neuroendocrine tumor	N/E			N/E		
Clinical stage						
I	1.00			1.00		
II	2.03	(0.84–4.94)	0.117	1.94	(0.78–4.78)	0.151
III	2.92	(1.26–6.74)	0.012	2.50	(1.06–5.89)	0.036
IV	7.79	(3.41–17.8)	<0.001	5.99	(2.57–13.96)	<0.001
Distant metastasis site						
Bone	2.53	(1.04–6.17)	0.041	2.56	(1.00–6.57)	0.050
Liver	2.40	(1.71–3.38)	<0.001	1.34	(0.92–1.96)	0.131

ECOG: 0 = fully active, 1 = restricted in strenuous activity, 2 = ambulatory and capable of self-care but unable to work, 3 = limited self-care, 4 = completely disabled. Clinical stage was assigned according to the Tumor–Node–Metastasis (TNM) staging system. TNM, Tumor–Node–Metastasis staging system, based on primary tumor extent (T), regional lymph node involvement (N), and distant metastasis (M). Clinical stage I indicates localized disease; stages II–III indicate progressively more extensive locoregional disease; and stage IV indicates metastatic disease. HR, Hazard Ratio; aHR, adjusted Hazard Ratio; 95%CI, 95% confidence interval; N/E, Not estimable, *the final multivariate model included ECOG, type of gastric cancer, clinical stage, distant metastasis (bone and liver) age and sex.

### 3.5 Sensitivity analysis

We found no evidence of meaningful bias attributable to ECOG missing data or to immortal time bias (Supplementary Table 1). aHR obtained from imputed datasets were similar to those obtained in the primary analysis with relative variations lower than 15% for all characteristics but ECOG 2 where aHR from imputed datasets was 2.88 compared to 2.37 from main analysis. In a similar fashion, HR obtained from landmark analysis showed lower relative variations, except for ECOG 4 (aHR = 4.04 vs. aHR = 7.03) and distant metastasis to the bone (aHR = 1.51 vs. aHR = 2.56), which due to small sample sizes (*n* = 4 and *n* = 7, respectively) and early deaths (<30 days) showed higher relative changes in their estimated HRs, that implied no clinical difference in interpretation.

Given the distinct biological and therapeutic profile of gastric stromal tumors, we performed an additional sensitivity analysis to assess the impact of histologic heterogeneity. Stromal tumors represented 18 patients (4.6%) in the analytic sample. Compared with adenocarcinoma, stromal tumors were associated with significantly lower adjusted median total direct costs (Int$ 32.7 k [95% CI: 3.7 k–61.7 k] vs. Int$ 62.7 k [95% CI: 56.6 k–68.8 k]; adjusted median difference: – Int$ 30.0 k [95% CI: −59.8 k to −0.2 k]; *p* = 0.048), after adjustment for length of follow-up, clinical stage, and Charlson comorbidity index. In survival analyses, stromal tumors were also associated with lower mortality compared with adenocarcinoma (HR 0.25; 95% CI: 0.08–0.77; *p* = 0.016). These findings are consistent with the expected clinical distinctiveness of this subgroup and support interpreting the main findings primarily in relation to the predominant adenocarcinoma.

## 4 Discussion

This study provides real-world evidence of the clinical and economic burden of gastric cancer in a tertiary hospital in a middle-income country. We observed a median overall survival of 30.7 months (95% CI: 22.7–38.7), with a Kaplan–Meier estimated 5-year overall survival probability of 40.0%. The estimated 5-year cumulative mean direct cost of care was Int$ 161.2 k (95% CI: 92.0 k–230.4 k). Patients diagnosed at early stages experienced substantially better survival (OS 76.3% for stage I), whereas advanced disease was associated with markedly higher cumulative costs, particularly among patients with stage IV disease, demonstrating that delayed diagnosis simultaneously worsens prognosis and increases financial pressure on the healthcare system.

Previous Colombian studies on OS in patients with gastric cancer have reported heterogeneous results. The population-based cancer registry of the city of Cali documented a five-year survival of 21.3% (95% CI: 19.2–23.6) for 2013–2017 (13), whereas a hospital-based study at Fundación Valle del Lili, a high-complexity hospital in the city of Cali, reported a five-year OS of 39.9% (95% CI 35.3–44.5) (14), a figure nearly identical to our estimate of 40%. In Bucaramanga, de Vries et al. reported a considerably lower five-year OS of 11% (15), highlighting marked regional disparities and uneven access to specialized services. Our findings align with those observed in high-complexity referral centers, suggesting that the concentration of oncologic expertise in tertiary hospitals substantially improves patient outcomes.

In other Latin American countries, studies have reported a five-year OS in the range of 10 to 41%, with Honduras reporting 9.9% and a median survival of only 4.8 months (16), Costa Rica reaching 40.6% (17), and Chile 16.7% (17). This variability reflects differences in healthcare infrastructure, access to oncologic treatments, and diagnostic capacity. A direct comparison of OS reported from different countries is not possible because of relevant methodological differences; however, Colombia ranks among the countries with the highest gastric cancer mortality rates in Latin America and the Caribbean, suggesting a substantial burden of advanced disease at diagnosis in the national context (18, 19).

Studies from different continents have reported five-year OS rates ranging from 68.9% in South Korea and 60.3% in Japan (17) to 33.1% in the United States (CONCORD-3 study), 19.9% in Denmark, and 35.4% in Austria (17). A Japanese series of surgically resected patients reported an overall survival of 71.1% (20), with stage-specific survivals of 91.5% (IA), 70.6% (II), 53.6% (IIIA), and 16.4% (IV). This disparity is largely attributed to national screening programs that identify 60–70% of cases at early stages in Asia (21), in contrast to the Latin American pattern, in which 73–90% are diagnosed at advanced stages (13). A recent analysis from China documented dramatic improvements in survival for stage III (from 60.3 to 70.2%) and stage IV (from 14 to 29%) between 2008 and 2012 and 2018–2022 (22), demonstrating that early detection and therapeutic advances can substantially modify prognosis.

To the best of our knowledge, there are no previous studies reporting cumulative costs of care, adjusting for censoring and deaths; however, our direct cost of care analysis reveals patterns consistent with the global literature on observed costs. Using data from Espinosa et al., 2024 (23) on the concentration, distribution, and persistence of health spending in Colombia—converted to 2023 international dollars (Int$) for comparability with our estimates—the 5-year mean cumulative direct cost of care for the gastric cancer patients in our study (Int$ 161,2 k) is approximately 14 times higher than the average per-capita healthcare expenditure for neoplasms among Colombians aged ≥60 years enrolled in the contributory scheme. On the other hand, the 5-year mean cumulative direct cost of care is equivalent to 7.2 times GDPs per capita (PPP), corresponding to an annualized ratio of 1.44 times GDP per capita, PPP (24).

The identified direct costs of International $49,000 annually were appropriate for an upper-middle-income setting when adjusted for purchasing power parity. In China, Zhang et al. reported average costs of USD 9,899, with 91.2% attributable to direct medical expenditures (25), whereas studies from the United States documented average monthly costs of USD 10,653 for incident cases versus USD 571 for controls (26), and a lifetime Medicare cost of USD 70,808 for advanced gastric cancer, with the average cost of care per hospitalization increasing from $21,710 in 2001 to $24,706 in 2011 (27). In patients with advanced metastatic gastric and esophageal cancer, the cost increased, with a mean all-cause cost of US$16,242 per patient per month for gastric cancer during first-line treatment (28).

In the United Kingdom, (29) conducted a cost–utility analysis reporting annual treatment costs of €22,434 – €23,498 by stage (29), with substantial increases in cost per quality-adjusted life year for advanced stages (€25,669 for stage IV versus €8,335 for stage I). This pattern reinforces that curative treatment is four to five times more cost-effective than palliative care (29), with direct implications for early detection policy. In Canada, the costs for metastatic gastric cancer treatment ranged from USD 34,002 to USD 72,778 depending on the treatment strategy over a 26-month horizon (30).

The cost distribution in our study (46% inpatient and 26% outpatient) mirrors international patterns, in which hospital-based care predominates (50–65%) (29, 31). In advanced gastric cancer, specific metastatic patterns such as bone involvement have been associated with increased healthcare utilization and costs due to pain management needs, bone-targeted therapies, and higher hospitalization rates (32).

The finding that 71.1% of patients in our study were diagnosed at stages III–IV is consistent with the national and regional patterns. Colombia’s High-Cost Diseases Fund has reported that 50.5% of new cases are diagnosed at advanced stages and 29.7% have unknown classification (33), while across Latin America, this figure approaches ~90%. In Mexico, 72.6% of patients have been documented at stage IV with a median interval of 4.21 months from symptom onset to diagnosis (34), and among younger patients (<30 years), this proportion reaches 83% (35), reflecting diagnostic delays attributable to low clinical suspicion in this age group.

The high proportion of advanced-stage diagnoses observed in our study indicates structural inefficiency in the cancer care pathway (36). International evidence demonstrates that organized endoscopic screening programs can reduce mortality by 40% (37). Our findings suggest that in Colombia, late presentation not only reduces survival but also increases cumulative healthcare expenditure. Consequently, early detection strategies can simultaneously improve health outcomes and reduce long-term costs, representing a potential high-value intervention for the healthcare system, despite the initial investment required to expand the diagnostic capacity.

In our study, patients were diagnosed with stage III–IV disease (36.3% stage III and 34.8% stage IV), whereas population-based screening programs in Asia largely invert this distribution, with a higher proportion of early-stage diagnoses. This contrast highlights a major public health concern. International evidence shows that biennial endoscopic screening in at-risk populations reduces mortality by 40–47% and is cost-effective when targeted to high-incidence regions (21). Colombia meets the epidemiological criteria to justify a national screening program; however, its implementation faces substantial challenges. The current endoscopic capacity in Latin America (2.5 per 100,000 population) is 14-fold lower than that in high-income countries (38), requiring major investments in infrastructure and workforce training.

A major strength of this study is the estimation of cumulative costs using methods that account for censoring and death rates. In oncology, early mortality and heterogeneous follow-up frequently bias the conventional cost estimates based on crude averages. By applying the Bang and Tsiatis estimator (12), our study provides time-dependent cost estimates that more accurately reflect the true financial burden of the disease over the course of treatment.

This study had several limitations. First, its retrospective design limits causal inference and is subject to documentation bias, missing data, and residual confounding. Although comorbidity burden was measured using the Charlson Comorbidity Index, detailed multimorbidity patterns and individual comorbid conditions were not modeled separately as cost predictors, which may introduce information and interpretation bias. Second, generalizability is limited because this was a single-center study, and selection bias is possible since patients treated at a tertiary referral hospital may differ from those managed in other settings or from patients who never access specialized oncologic services. In this context, gastric stromal tumors were rare among the selected patients (18 cases; 4.6%) and may reflect referral patterns in a tertiary hospital; therefore, findings related to this subgroup should be interpreted cautiously, and the main interpretation of the study remains driven by the predominant adenocarcinoma population. Third, clinical stage, treatment intent, metastatic involvement, and healthcare utilization were abstracted from routine clinical and administrative records, which may have led to misclassification or incomplete documentation. Finally, the economic evaluation was conducted from the healthcare provider perspective and included only direct medical costs billed within the institution, excluding costs incurred elsewhere, indirect costs, and non-medical expenses borne by patients and caregivers.

## 5 Conclusion

Gastric cancer imposes a substantial clinical and financial burden in middle-income settings, largely driven by late-stage diagnosis. Our findings suggest that delayed detection generates a dual penalty for the healthcare system, resulting in worse survival and higher cumulative costs of care. Policies aimed at earlier diagnosis and timely referral to specialized services may represent a high-value strategy capable of improving outcomes while reducing long-term resource utilization. These results provide locally generated real-world evidence to support cancer control planning and future economic evaluations in Colombia and in similar health systems.

## Data Availability

The raw data supporting the conclusions of this article will be made available by the authors, without undue reservation.
