# Supplementary material for: Estimated five-year survival and direct healthcare costs of adult patients with gastric cancer: real-world evidence from a tertiary hospital in Colombia
Source: Front Public Health. 2026 Jun 18;14:1820842. doi: 10.3389/fpubh.2026.1820842 (PMC13329146; doi:10.3389/fpubh.2026.1820842)
Supplement: Supplementary file 1 [file Table_1.docx]

**Supplemental Table 1.** Sensitivity analyses of potential bias attributable to ECOG missing data and potential immortal time bias

|  | **Complete case analysis** | | **Chained equations MI analysis** | | | **MI Landmark (30 days) analysis** | | |
| --- | --- | --- | --- | --- | --- | --- | --- | --- |
|  | **aHR*** | **p-value** | **aHR*** | **p-value** | **variation** | **aHR*** | **p-value** | **variation** |
| ECOG Performance Status Scale |  |  |  |  |  |  |  |  |
| 0 | 1.00 |  | 1.00 |  |  | 1.00 |  |  |
| 1 | 1.44 | 0.057 | 1.55 | 0.026 | 7.4% | 1.50 | 0.044 | 4.1% |
| 2 | 2.37 | 0.001 | 2.88 | <0.001 | 21.7% | 2.56 | 0.001 | 8.0% |
| 3 | 3.88 | <0.001 | 4.02 | <0.001 | 3.7% | 3.66 | <0.001 | -5.7% |
| 4 | 7.03 | <0.001 | 6.94 | 0.001 | -1.2% | 4.04 | 0.07 | -42.6% |
| Type of gastric cancer |  |  |  |  |  |  |  |  |
| Adenocarcinoma | 1.00 |  | 1.00 |  |  | 1.00 |  |  |
| Squamous-cell carcinoma | N/E |  | N/E |  |  | N/E |  |  |
| Stromal tumor | 0.28 | 0.03 | 0.28 | 0.033 | 1.5% | 0.28 | 0.033 | 1.4% |
| Neuroendocrine tumor | N/E |  | N/E |  |  | N/E |  |  |
| Clinical stage |  |  |  |  |  |  |  |  |
| I | 1.00 |  | 1.00 |  |  | 1.00 |  |  |
| II | 1.94 | 0.151 | 2.12 | 0.105 | 9.3% | 1.86 | 0.184 | -4.3% |
| III | 2.5 | 0.036 | 2.76 | 0.02 | 10.6% | 2.55 | 0.033 | 2.0% |
| IV | 5.99 | <0.001 | 6.15 | <0.001 | 2.6% | 5.99 | <0.001 | 0.1% |
| Distant metastasis site |  |  |  |  |  |  |  |  |
| Bone | 2.56 | 0.05 | 2.92 | 0.044 | 14.1% | 1.51 | 0.571 | -41.0% |
| Liver | 1.34 | 0.131 | 1.41 | 0.08 | 5.2% | 1.30 | 0.197 | -2.7% |

**MI:** Multiple imputation, **aHR:** adjusted Hazard Ratio, **95%CI:** 95% confidence interval, **N/E:** Not estimable, ***:** the final multivariate model included ECOG, type of gastric cancer, clinical stage, distant metastasis (bone and liver) age and sex.
